# Ventilating Chambers

**Published:** 1870-09

**Authors:** 


### VENTILATING CHAMBERS.

If a lamp or candle, or a very little fire is kept burning in a fire-place at night, a draught is created up the chimney, by which the foulest air in the room is carried out with great rapidity.

In all cases where there is a fire-place in a chamber, it should by all means be kept open.

As a general rule, the windows of a chamber should be left open more or less, according to the outside temperature. If the sash is arranged to be let down from the top, as well as hoisted from the bottom, then both should be open. But some windows are made to hoist from the bottom only; in this or other cases, where the draught from that part may be inconvenient, take a board, as long as the window sash is broad, and itself two or more inches broad, raise the sash, put this board in and bring down the sash upon it, then there is an aperture near the joinings of the lower and upper sash through which a steady stream of pure air flows from without into the room, in the direction of the ceiling, and by the time it gets to the sleeper it is warmed; thus, also, is a direct draught upon the person avoided. As a third of our existence is passed in our chambers, we should do all that is practicable in these little ways, to give ourselves a pure atmosphere, resulting in good sleep, and a good appetite for breakfast.

